# Data-Mining Techniques: A New Approach to Identifying the Links among Hybrid Strains of *Pleurotus* with Culture Media

**DOI:** 10.3390/jof7100882

**Published:** 2021-10-19

**Authors:** Fabricio Guevara-Viejó, Juan Diego Valenzuela-Cobos, Purificación Vicente-Galindo, Purificación Galindo-Villardón

**Affiliations:** 1 Facultad de Ciencias e Ingeniería, Universidad Estatal de Milagro (UNEMI), Milagro 091050, Ecuador; jguevarav@unemi.edu.ec (F.G.-V.); juan_diegova@hotmail.com (J.D.V.-C.); 2 School of Medicine, Universidad Espíritu Santo, Guayaquil 092301, Ecuador; 3 Department of Statistics, University of Salamanca, 37008 Salamanca, Spain; purivg@usal.es; 4 Institute for Biomedical Research of Salamanca (IBSAL), 37006 Salamanca, Spain; 5 Centro de Investigación Institucional, Universidad Bernardo O’Higgins, Av. Viel 1497, Santiago 8320000, Chile

**Keywords:** circular economy, data-mining techniques, mushroom market

## Abstract

In this study, a data set of mycelial and cultural characteristics of hybrid strains of *Pleurotus ostreatus* and *Pleurotus djamor* were analyzed using three data-mining techniques: the K-medoids clustering algorithm, PCA biplot and the association rules algorithm. The characteristics evaluated were as follows: maximum velocity; lag phase; biomass; and exopolysaccharides content in the cultivation of 50 hybrid strains of *Pleurotus ostreatus* and 50 hybrid strains of *Pleurotus djamor.* Different mixtures of culture media were used to supplement Ecuadorian agricultural products. Data of the parameters obtained in the experimental methods were grouped into four clusters, obtaining a presentation of the hybrid strains of *Pleurotus* with a higher relation to each characteristic measured. Data-mining tools showed the hybrid strains cultivated on solid-culture media (M1 = malt extract agar and rice flour) and liquid-culture media (L1 = maltose, yeast extract and rice flour) presented the highest mycelial and cultural characteristics. These results are good indicators to improve the industrial production of edible fungi by using rice flour in the cultivation, contributing to the mushroom market and circular economy.

## 1. Introduction

Data mining is the process of extracting understandable and useful information from big data, with its main objective being to find hidden or implicit information which is not possible to obtain by methods of conventional statistics. The beginning of the mining process is formed generally by records from operational databases or data warehouses [1]. Data mining is an extraction process of information that involves the search for behavior patterns that are hidden at first glance among large amounts of information [2]. There are various algorithms and techniques that describe the interesting relationship between different attributes, such as the K-medoids clustering algorithm, PCA biplot and the association rules algorithm.

In the agricultural sector, where agribusinesses must make countless decisions every day, considering various intricate complexities and factors which influence them, it is necessary that an accurate estimation of the yield of the crops is involved in agricultural planning. Data-mining techniques are a necessary approach to achieve practical and effective solutions to this problem; therefore, agriculture is an obvious target for big data [3]. Agriculture in Ecuador is one of the main sectors that maintains economic dynamics and supplies raw materials to the food industry, promoting the country’s food security and sovereignty [4]. The main effects of agriculture are to reduce hunger and malnutrition, improve living conditions, increase income and generate employment for vulnerable groups living in poverty [5]. The main Ecuadorian export products that have gained global popularity over time are bananas, cocoa and flowers [6]. However, there are some agricultural products that have presented economic losses for farmers, such as rice and soybeans [7,8], because these products are offered at cheap prices in countries near to Ecuador. Therefore, the official prices of these products are not respected by the industry [9], and in some cases, the rice and soybeans are used as animal feed.

*Pleurotus* genus is one of the most commercialized groups of mushrooms in the world, due to the ease of its cultivation, great economic potential, and flavor [10,11]. Moreover, it only requires tropical and subtropical climates to produce fruit bodies [12]. However, the production of *Pleurotus* in small-scale companies presents some problems, such as product contamination and a difficulty in obtaining good-quality spawns [13]. The development of hybrid strains allows for the improvement of commercial attributes by reducing the incubation time, using different agricultural residues for the cultivation of the fungi and making it more adaptable to climatic conditions [14,15]. A number of studies have focused on the biotechnological development of fungi strains and inoculum, such as the use of solid-culture media with the supplementation of agricultural products to improve the growth speed, and the influence of nutrients from the inoculum in liquid media to increase the production of biomass and exopolysaccharides [16,17,18]. The nutritional and environmental requirements of the edible fungi strains show direct relationships with the productivity parameters after cultivation [19]. The production of edible fungi is a profitable business due to the use of low-cost agricultural products and food waste [20].

The main goal of this study was to use data-mining techniques such as the K-medoids clustering algorithm, PCA biplot and the association rules algorithm to identify the hybrid strains of *Pleurotus ostreatus* and *Pleurotus djamor,* using culture media supplemented with the agricultural products of rice and soybeans that obtained the highest values in mycelial and cultural characteristics.

## 2. Materials and Methods

### 2.1. Mushroom Strains

For this study, 50 hybrid strains of Pleurotus ostreatus (PO) and 50 hybrid strains of Pleurotus djamor (PD) were used. The strains were hybrids obtained through the pairing of compatible neohaplonts of Pleurotus djamor or monokaryons of Pleurotus ostreatus. The neohaplonts were obtained by chemical dedikaryotization, and the Pleurotus strains were maintained on MEA dishes and deposited at the fungal collection of the Research and Development Laboratory of Ecuahidrolizados.

### 2.2. Chemical Dedikaryotization

The mycelium of *Pleurotus* spp. on MEA dishes was divided into four parts and put into a blender (Model N.4237, Mark: Marnie). Then, it was homogenized with 50 mL of sterile water for 60 s, and 25 µL homogenate was inoculated in 100 mL flasks with 50 mL dedikaryotization solution (20% of anhydrous glucose and 20% of peptone) and incubated at 28 °C. When the mycelium growth was noticeable in the flasks with the dedikaryotization solution, the flasks were homogenized with 50 mL sterile distilled water for 60 s, and 25 µL of this homogenate was inoculated on MEA plates and incubated at 28 °C until colonies were formed. Growing colonies were observed under the microscope and identified as neohaplonts, characterized by the absence of clamp connections [21,22].

### 2.3. Identification of Neohaplonts’ Compatibility Types and Production of Reconstituted Strains

To identify the two types of neohaplonts (mycelium with an absence of clamp connections) in the parental strains of *Pleurotus ostreatus* or *Pleurotus djamor*, a monokaryotic component of *Pleurotus ostreatus* was randomly selected and paired in the MEA dishes with all remaining neohaplonts in the *Pleurotus ostreatus*. The same product was realized with the monokaryons in the *Pleurotus djamor*. The dikaryotic mycelium was characterized by the presence of clamp connections and verified under the microscope 10(x) [23]. Authors have reported that this method for the production of hybrid strains presented a high degree of polymorphism among the hybrid strains and the parental strains. The high degree of polymorphism indicated the genetic diversity that existed between the strains [24].

### 2.4. Preparation of Mixtures of Culture Media

Hybrid strains of *Pleurotus* were cultivated using two mixtures of culture media:

M1 = 18 g of malt extract, 15 g of bacteriological agar and 20 g of rice flour in 1 L of distilled water.

M2 = 18 g of malt extract, 15 g of bacteriological agar and 20 g of soybean flour in 1 L of distilled water.

The dishes with the solidified media were incubated at 28 °C for 24 h to check for sterility.

### 2.5. Determination of Mycelial Area

The diameter of the colony was measured daily until the mycelium colonized the Petri dishes with M1 and the Petri dishes with M2, as seen in Equation (1) [25]:

(1) $$A\;=\frac{\pi d^{2}}{4}\;$$

Equation (1). Mycelial Area.

### 2.6. Mathemical Model

To calculate the mycelial growth speed (μ_max_) and the lag time (λ) on the Petri dishes with M1 and the Petri dishes with M2, the mycelial area was fitted to the Baranyi Model, as seen in Equation (2) [26]:

(2) $$y\left(t_{\max}\right)\;=\frac{y_{\max}+\;\ln\;((-1+{\;e}^{{\text{µ}}_{\max}\;\lambda \;}+e^{{\text{µ}}_{\max}t})}{\left(-1+e^{{\text{µ}}_{\max}t}\right)+e^{\left({\text{µ}}_{\max}\;\lambda \;+{\;y}_{\max}-y_{0}\right)})}\;$$

Equation (2). Baranyi Model.

### 2.7. Biomass Production

Two discs of mycelium (5.5 mm) from the hybrid strains of *Pleurotus ostreatus* (PO) and *Pleurotus djamor* (PD) were cut from the edge of the Petri dishes with solid liquid M1, and were then inoculated in 100 mL of the solution of liquid culture (L1 = 1 L of distilled water with maltose (40 g L^−1^), yeast extract (3 g L^−1^) and rice flour (2 g L^−1^)). Furthermore, two discs of mycelium from the hybrid strains of *Pleurotus* were cut from the edge of the plates with solid liquid M2, and were then inoculated in 100 mL of the solution of liquid culture (L2 = 1 L of distilled water with maltose (40 g L^−1^), yeast extract (3 g L^−1^) and soybean flour (2 g L^−1^)).

All production studies were carried out at 28 °C and 150 rpm in a shaking incubator for 7 days. Cellular biomasses were separated by using a 20,000 rpm centrifuge at 4 °C, and were then washed from the sieve with distilled water, filtered through Whatman #1 filter paper, and dried to a constant weight at 80 °C [27].

### 2.8. Exopolysaccharides Production

The culture broth and the water used to wash the biomass off the sieves were filtered through Whatman #1 filter paper and evaporated to 50 mL at 80 °C using a heating plate. This reduced volume was added to 150 mL of ethanol (98%), in order to precipitate the exopolysaccharides (EPS). The precipitated exopolysaccharides was filtered out and dried to constant weight at 40 °C [28,29].

### 2.9. Statistical Analysis

The mycelial characteristics of maximum velocity and lag phase, as well as the cultural characteristics of biomass and exopolysaccharides content, were measured in triplicates (for each hybrid strain growing on the different culture media). The data-mining techniques (K-medoids, PCA biplot and association rules) were realized using R software version 4.1.1.

#### 2.9.1. K-Medoids Clustering

The K-medoids algorithm is a method of unsupervised classification [30]. The sequence of the K-medoids algorithm is as follows:

1. Select a comparison function between objects. For example, if we are dealing with qualitative variables, we usually use the Euclidean distance;

$$\|X_{i}-X_{j}\|\;=\sqrt{\overset{p}{\underset{k\;=1}{\sum }}{\left(X_{\mathrm{ik}}-X_{\mathrm{jk}}\right)}^{2}}$$

2. Calculate the Global Matrix of similarity or difference, that is, the distance matrix;
3. Select the K-farthest patterns as initial attractors;
4. Calculate and store the similarity or difference between each pattern and each of the K-attractor objects;
5. Partition the space into groups, assigning each pattern to the closest attractor group;
6. Calculate, for each defined group, its medoid;
7. Consider the newly calculated medoids as new attractor patterns;
8. Return to step (4);
9. Terminate when the set of medoids is identical to that of the previous iteration.

The K-medoids algorithm has a mechanism for grouping (by partitioning) objects in any representation space. By calculating medoids instead of centroids, the K-medoids algorithm converges faster to the only possible global solution in that representation space, and with that set of objects.

#### 2.9.2. PCA Biplot

A biplot approximates a matrix performed without making assumptions about the underlying probabilistic distributions that provide the geometric structure of the data graphically, showing the variability of the set of individuals and variables. The prefix *bi* refers to the representation of simultaneous rows and columns of the matrix.

Theoretically, in a biplot, a rectangular matrix Y of order (*nxp*) and rank r, by another of rank q (q < r), has its decomposition into singular values (DVS) given by:

Y≅UΣV ′

where U and V are matrices of orthonormal singular vectors such that U′U = V′V = I (where I is the identity matrix) and ∑ is a diagonal matrix containing the α_k_ greatest singular values.

To guarantee the representation is necessary, a factorization such as: Y ≅ (UΣ^S^) (Σ^1−S^ V’) = AB‘, with A and B being the matrices that contain the coordinates of the (*n + p*) vectors or markers rows a_i_ and columns b_j_ to use over the graphic (i = 1, …, *n*; j = 1, …, *p*) [31].

#### 2.9.3. Association Rules

The mining of association rules is considered to be the main task in data mining. An association rule expresses an interesting relationship between different attributes [32].

An association rule implies the form X ⇒ Y, where X and Y are itemsets; X is the body and Y is the head. A rule can be evaluated by two measures, called confidence and support. The support for the association rule X ⇒ Y is the percentage of transactions that contain both itemset X and Y among all transactions. The confidence for the association rule X ⇒ Y is the percentage of transactions that contain an itemset Y among the transactions that contain an itemset X. Support represents the usefulness of the discovered rules, and confidence represents the certainty of the rules [33].

## 3. Results and Discussion

The focus of this research was to identify the links among hybrid strains of *Pleurotus* with the culture media that obtained the highest mycelial and cultural characteristics.

The mycelial characteristics measured were maximum velocity and lag phase, whereas the cultural characteristics determined were biomass and exopolysaccharides content.

The numeration of the strains followed this distribution:

Hybrid strains 1–50 of *Pleurotus ostreatus* or *Pleurotus djamor* cultivated on (M1 = malt extract agar with rice flour and L1 = maltose, yeast extract and rice flour);

Hybrid strains 51–100 of *Pleurotus ostreatus* or *Pleurotus djamor* cultivated on (M2 = malt extract agar with soybean flour and L2 = maltose, yeast extract and soybean flour).

### 3.1. Clustering K-Medoids Algorithm for Mycelial and Cultural Characteristics of the Hybrid Strains of Pleurotus

Table 1 indicates the distribution of the four clusters for the mycelial and cultural characteristics of the hybrid strains of *Pleurotus ostreatus* cultivated on solid culture (M1 and M2) and on liquid culture (L1 and L2). The size of cluster 1 is 33, the size of cluster 2 is 17, the size of cluster 3 is 16, and the size of cluster 4 is 34. The hybrid strains of *Pleurotus ostreatus* growing on solid culture (M2) and on liquid culture (L2) belonged to cluster 1 and cluster 2, whereas the hybrid strains of *Pleurotus ostreatus* cultivated on culture media (M1) and on liquid media (L1) belonged to cluster 3 and cluster 4. We found that the hybrid strains of *Pleurotus ostreatus* cultivated on solid culture (M1) and on liquid culture (L1) belonging to cluster 1 (yellow) presented the highest mycelial and cultural characteristics, and the hybrid strains of *Pleurotus ostreatus* grown on solid culture (M2) and on liquid culture (L2) belonging to cluster 4 (yellow) also presented the highest mycelial and cultural characteristics. The K-medoids clustering algorithm indicated that the number of strains cultivated on the different culture media presented the highest mycelial and cultural characteristics.

The yellow represents the number of *Pleurotus ostreatus* strains growing on the solid culture and the liquid media that showed the highest mycelial and cultural characteristics.

The culture medium M1 contained 18 g of malt extract, 15 g of bacteriological agar and 20 g of rice flour in 1 L of distilled water. The culture medium L1 contained 40 g of maltose, 3 g of yeast extract and 2 g of rice flour in 1 L of distilled water.

The culture medium M2 contained 18 g of malt extract, 15 g of bacteriological agar and 20 g of soybean flour in 1 L of distilled water. The culture medium L2 contained 40 g of maltose, 3 g of yeast extract and 2 g of soybean flour in 1 L of distilled water.

Table 2 presents the distribution of the four clusters for the mycelial and cultural characteristics of the hybrid strains of *Pleurotus djamor* grown on culture media (M1 and M2) and on liquid media (L1 and L2). The size of cluster 1 is 27, the size of the cluster 2 is 18, the size of the cluster 3 is 30, and the size of the cluster 4 is 25. The hybrid strains of *Pleurotus djamor* were grown on solid culture (M1) and on liquid culture (L1) belonging to cluster 1, cluster 2 and cluster 3, whereas the hybrid strains of *Pleurotus djamor* cultivated on culture medium (M2) and on liquid medium (L2) belonged to cluster 3 and cluster 4; this result indicated that the hybrid strains of *Pleurotus djamor* grown on solid culture (M1) and on liquid culture (L1) belonging to cluster 1, cluster 2 and cluster 3 (yellow) presented the highest mycelial and cultural characteristics, whereas the hybrid strains of *Pleurotus djamor* cultivated on solid culture (M2) and on liquid culture (L2) belonging to cluster 3 (yellow) presented the highest mycelial and cultural characteristics. Data points are normally distributed, and clusters may vary in size with maximum data points and minimum data points.

The variability in the characteristics of the hybrid strains (the high rates of invasion in substrates, and high productivities) was due to the separation of the nuclei during the formation of the monokaryons and their subsequent union during the formation of the dikaryon (hybrid strains) [34]. The K-medoids clustering algorithm allowed us to determine the presence of four clusters indicating the relations among the hybrid strains of *Pleurotus ostreatus* and the hybrid strains of *Pleurotus djamor* with the mycelial characteristics of maximum velocity and phase lag, and the cultural characteristics of biomass content and exopolysaccharides.

The yellow indicates the number of *Pleurotus djamor* strains cultivated on solid culture and liquid media that showed the highest mycelial and cultural characteristics.

The culture medium M1 contained 18 g of malt extract, 15 g of bacteriological agar and 20 g of rice flour in 1 L of distilled water. The culture medium L1 contained 40 g of maltose, 3 g of yeast extract and 2 g of rice flour in 1 L of distilled water.

The culture medium M2 contained 18 g of malt extract, 15 g of bacteriological agar and 20 g of soybean flour in 1 L of distilled water. The culture medium L2 contained 40 g of maltose, 3 g of yeast extract and 2 g of soybean flour in 1 L of distilled water.

### 3.2. PCA Biplot Algorithm for Mycelial and Cultural Characteristics of the Hybrid Strains of Pleurotus

**Figure 1:**
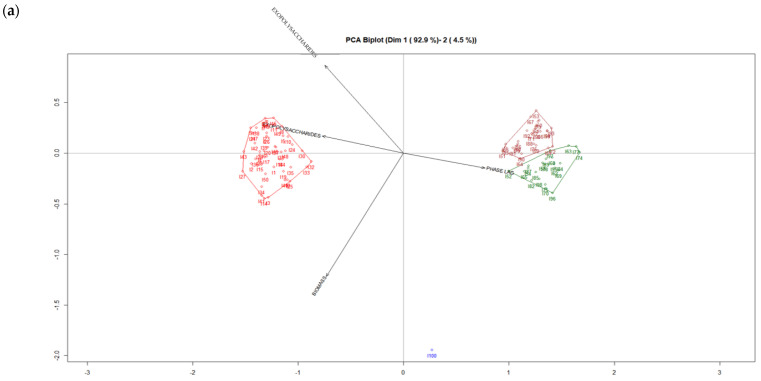

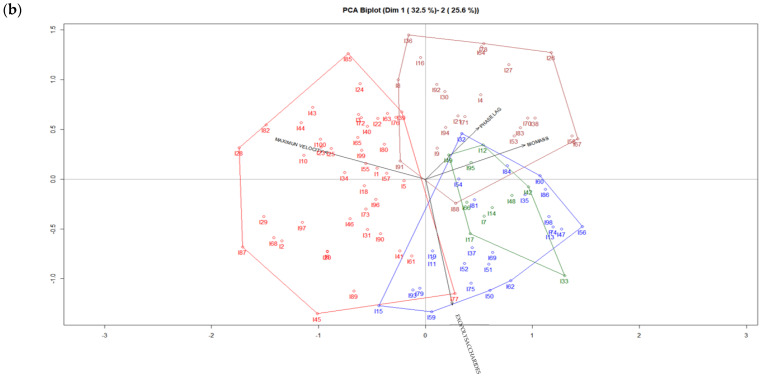
(**a**) PCA biplot for the mycelial and cultural characteristics of the hybrid strains of *Pleurotus ostreatus* cultivated on solid culture M1 and M2, and liquid culture L1 and L2, (**b**) PCA biplot for the mycelial and cultural characteristics of the hybrid strains of *Pleurotus djamor* cultivated on solid culture M1 and M2 and liquid culture L1 and L2.

Figure 1 presents the factorial graph of the plane 1-2 (PCA biplot). Figure 1a shows the accumulated inertia amounts to 97.4%, whereas Figure 1b presents the accumulated inertia amounts of 58.1% In addition, the clusters were calculated using the biplot coordinates, and the overview of the clusters was based on four variables. We can see in Figure 1a the important differences between the clusters; cluster 1 (red) shows the presence of 50 hybrid strains of *Pleurotus ostreatus* growing on solid culture (M1) and on liquid culture (L1) with a higher relation to the following parameters: maximum velocity, biomass and exopolysaccharides content. On the other hand, cluster 2 (brown), cluster 3 (green) and cluster 4 (blue) indicate the presence of 50 hybrid strains of *Pleurotus ostreatus* cultivated on solid culture (M2) and on liquid culture (L2) with a higher relation to the lag phase. Furthermore, Figure 1b demonstrates that there are differences between the clusters; cluster 1 (red) indicates the presence of 42 hybrid strains of *Pleurotus djamor* growing on the two culture media and in the liquid culture (L1 and L2) with a higher relation to the maximum velocity and exopolysaccharides content, whereas cluster 2 (brown) shows the presence of 22 hybrid strains of *Pleurotus djamor* cultivated on the culture media (M1 and M2) and on the liquid culture (L1 and L2) with a higher relation to the lag phase and biomass content. Cluster 3 (blue) and cluster 4 (green) indicate the presence of 36 hybrid strains of *Pleurotus djamor* growing on the two culture media and on the two liquid cultures with a higher relation to exopolysaccharides content.

Maltose is the most suitable carbon source for both mycelial biomass and exopolysaccharides content, whereas yeast extract is the favorable nitrogen source for both mycelial biomass and EPS production [35]. The use of submerged culture in the cultivation of edible fungi represents an alternative method of the rapid and efficient production of biomass and the production of exopolysaccharides (EPS) [36]. The main interest in the production of EPS by fungi is due to its biological and pharmacological activities, such as its immunostimulation, antitumor and hypoglycemic qualities [37]. The use of culture media supplemented with rice flour allowed the strains to colonize the substrate in a short amount of time, in comparison with the culture media supplemented with soybean flour. The solid culture M1 (malt extract agar with rice flour) and liquid culture L1 (maltose, yeast extract and rice flour) can be used to obtain the highest mycelial and cultural characteristics in the growing of hybrid strains of *Pleurotus.*

### 3.3. Association Rules Algorithm for Mycelial and Cultural Characteristics of Hybrid Strains of Pleurotus

**Figure 2:**
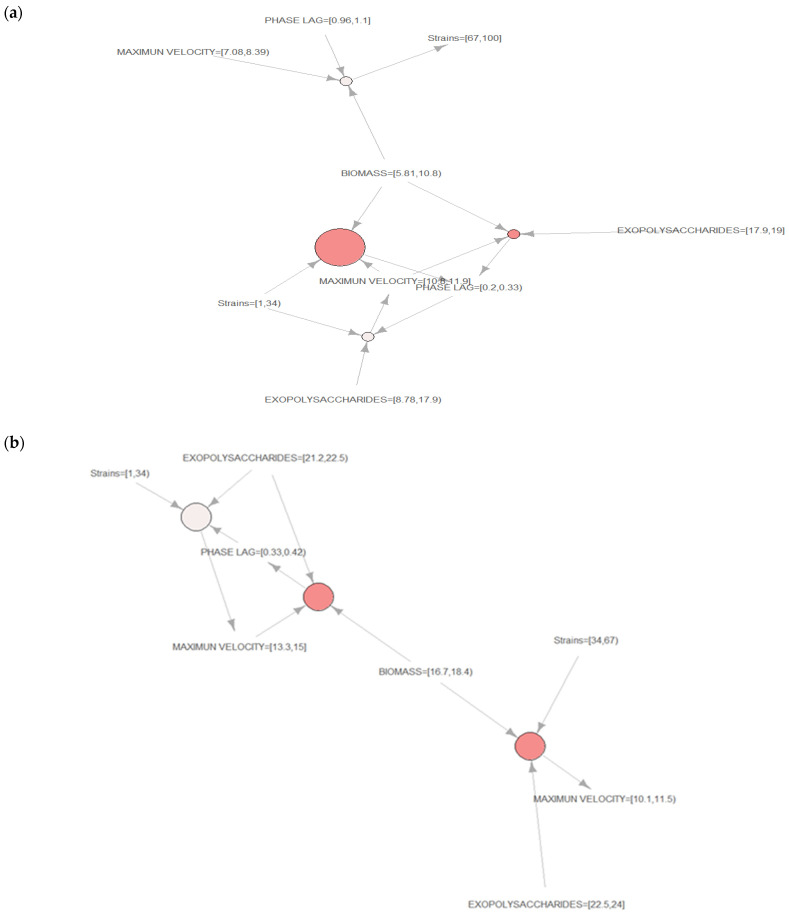
(**a**) Association rules algorithm for mycelial and cultural characteristics of hybrid strains of *Pleurotus ostreatus* cultivated on solid culture M1 and M2, and liquid culture L1 and L2, (**b**) Association rules algorithm for mycelial and cultural characteristics of hybrid strains of *Pleurotus djamor* cultivated on solid culture M1 and M2, and liquid culture L1 and L2.

Figure 2 presents the use of association rules to a data set of hybrid strains of *Pleurotus ostreatus* and *Pleurotus djamor* cultivated on solid culture (M1 and M2) and on liquid culture (L1 and L2). The association rules algorithm is a successful solution for extracting alternate rules, because it provides a complete picture of associations in a large data set.

Figure 2a shows a group of hybrid *Pleurotus ostreatus* strains (1 to 33) cultivated on solid culture (M1) and liquid culture (L1) with the following mycelial and cultural characteristics: maximum velocity between 10.8 and 11.9 h^−1^, lag phase from 0.2 to 0.33 h, biomass content ranging from 5.81% to 10.8% and exopolysaccharides between 8.78% and 17.9%. Moreover, it also presents a group of hybrid strains of *Pleurotus ostreatus* (67 to 100) growing on solid medium (M2) and liquid medium (L2) with the following mycelial and cultural characteristics: maximum velocity between 7.08 and 8.39 h^−1^, lag phase from 0.96 to 1.1 h, and biomass content ranging from 5.81% to 10.8%.

Figure 2b presents a group of hybrid strains of *Pleurotus djamor* (1 to 33) growing on solid culture (M1) and liquid culture (L1) with the following mycelial and cultural characteristics: maximum velocity from 13.3 to 15 h^−1^, lag phase between 0.33 and 0.42 h, biomass content ranging from 16.7% to 18.4%, and exopolysaccharides between 21.2% and 22.5%. It also shows a group of hybrid strains of *Pleurotus djamor* (34 to 66) with the following mycelial and cultural characteristics: maximum velocity from 10.1 to 11.5 h^−1^, biomass content ranging from 16.7% to 18.4%, and exopolysaccharides between 22.5% and 24%.

The mycelial and cultural characteristics of each strain can be used as a selection criterion for edible-mushroom-cultivation programs. The strains with the highest maximum velocity and lowest lag phases have the potential to obtain the highest commercial parameters, because they colonize the substrate in the least amount of time, while still allowing for the obtainment of the fruiting characteristics [38]. The optimal submerged culture conditions for maximum mycelial growth and exopolysaccharides production depend strongly on the type of substrates and fungal species [39].

The association rules algorithm allowed us to identify a link among a group of *Pleurotus ostreatus* and *Pleurotus djamor* hybrid strains. The solid and liquid culture media allowed us to obtain the highest mycelial characteristics of maximum velocity and phase lag, and the highest cultural characteristics of biomass content and exopolysaccharides.

## 4. Conclusions

The K-medoids clustering algorithm allowed us to determine the presence of four clusters that indicated the relation between the hybrid strains of *Pleurotus ostreatus* and *Pleurotus djamor,* with determined mycelial and cultural characteristics.

PCA biplot presented the specific hybrid strains of *Pleurotus ostreatus* and the specific hybrid strains of *Pleurotus djamor* cultivated on the different culture media (solid and liquid) with the characteristics measured.

The association rules algorithm identified the link between the hybrid strains of *Pleurotus ostreatus* and the hybrid strains of *Pleurotus djamor* with the solid culture (M1) and the liquid culture (L1) that obtained the highest mycelial and cultural characteristics.

## Figures and Tables

**Table 1:** Number of hybrid strains of *Pleurotus ostreatus* growing on different culture media belonging to each cluster using the clustering K-medoids algorithm.

Culture Media	Cluster 1	Cluster 2	Cluster 3	Cluster 4
M1 + L1	33	17		
M2 + L2			16	34

**Table 2:** Number of hybrid strains of *Pleurotus djamor* cultivated on different culture media belonging to each cluster using the clustering K-medoids algorithm.

Culture Media	Cluster 1	Cluster 2	Cluster 3	Cluster 4
M1 + L1	13	6	14	17
M2 + L2	14	12	16	8

## Data Availability

Not applicable.
